# Predicting Deep Learning Based Multi-Omics Parallel Integration Survival Subtypes in Lung Cancer Using Reverse Phase Protein Array Data

**DOI:** 10.3390/biom10101460

**Published:** 2020-10-19

**Authors:** Satoshi Takahashi, Ken Asada, Ken Takasawa, Ryo Shimoyama, Akira Sakai, Amina Bolatkan, Norio Shinkai, Kazuma Kobayashi, Masaaki Komatsu, Syuzo Kaneko, Jun Sese, Ryuji Hamamoto

**Affiliations:** 1 Cancer Translational Research Team, RIKEN Center for Advanced Intelligence Project, 1-4-1 Nihonbashi, Chuo-ku, Tokyo 103-0027, Japan; satoshi.takahashi.fy@riken.jp (S.T.); ktakazaw@ncc.go.jp (K.T.); norio.shinkai@riken.jp (N.S.); kazumkob@ncc.go.jp (K.K.); maskomat@ncc.go.jp (M.K.); 2 Division of Molecular Modification and Cancer Biology, National Cancer Center Research Institute, 5-1-1 Tsukiji, Chuo-ku, Tokyo 104-0045, Japan; rshimoya@ncc.go.jp (R.S.); akira.sakai@jp.fujitsu.com (A.S.); abolatka@ncc.go.jp (A.B.); sykaneko@ncc.go.jp (S.K.); sesejun@humanome.jp (J.S.); 3 Humanome Lab, 2-4-10 Tsukiji, Chuo-ku, Tokyo 104-0045, Japan

**Keywords:** lung cancer, multi-omics analysis, deep learning and machine learning

## Abstract

Mortality attributed to lung cancer accounts for a large fraction of cancer deaths worldwide. With increasing mortality figures, the accurate prediction of prognosis has become essential. In recent years, multi-omics analysis has emerged as a useful survival prediction tool. However, the methodology relevant to multi-omics analysis has not yet been fully established and further improvements are required for clinical applications. In this study, we developed a novel method to accurately predict the survival of patients with lung cancer using multi-omics data. With unsupervised learning techniques, survival-associated subtypes in non-small cell lung cancer were first detected using the multi-omics datasets from six categories in The Cancer Genome Atlas (TCGA). The new subtypes, referred to as integration survival subtypes, clearly divided patients into longer and shorter-surviving groups (log-rank test: *p* = 0.003) and we confirmed that this is independent of histopathological classification (Chi-square test of independence: *p* = 0.94). Next, an attempt was made to detect the integration survival subtypes using only one categorical dataset. Our machine learning model that was only trained on the reverse phase protein array (RPPA) could accurately predict the integration survival subtypes (AUC = 0.99). The predicted subtypes could also distinguish between high and low risk patients (log-rank test: *p* = 0.012). Overall, this study explores novel potentials of multi-omics analysis to accurately predict the prognosis of patients with lung cancer.

## 1. Introduction

Lung cancer is the most commonly diagnosed cancer worldwide and is the leading cause of cancer death. There were an estimated 2.1 million cases with 1.8 million deaths due to lung cancer in 2018 [1]. The incidence and mortality rates vary among regions; for example, the incidence rate is higher in Polynesia, Micronesia, North America, East Asia and Europe. According to estimates, the United States is likely to see more than 228,820 people newly diagnosed cases with more than 135,720 lung cancer deaths in 2020 [2]. Similarly, the incidence and the mortality rates are expected to rise in East Asia, including Japan [3].

There are two major types of lung cancers: small cell lung cancer (SCLC) and non-small cell lung cancer (NSCLC). The percentage of patients diagnosed with NSCLC (around 80–85%) is greater than SCLC (around 15–20%). NSCLC consists of three histological subtypes, adenocarcinoma (LUAD, around 40% of all lung cancer cases), squamous cell carcinoma (LUSC, around 25–30%) and large cell carcinoma (around 10–15%) [4]. It is evident with increasing reported studies that lung cancer represents a group of histologically and molecularly heterogeneous diseases even within the same histological subtype [5,6,7]. Hence, a new classification method, independent of histological subtypes and specific gene mutations, is required to be developed. To provide a better decision-making tool, this new classification method should be able to predict clinical outcomes using appropriate datasets or labels. Once an appropriate new classification system is established, treatment based on the classification could be possible and more appropriate cancer treatment could be provided to lung cancer patients.

The key to creating this desired classification platform is to use multi-omics data. There are two levels of omics data: single-level omics data and multi-omics data [8]. Single-level omics data consist of one data type or category. A representative example is a microarray analysis of gene expression. Medical images, when we treat them as data, are another example. Single-level omics data analysis is useful and may be able to predict the prognosis or treatment response in cancer patients. However, single-level omics data analysis has a limitation. In other words, one type of data may not be able to appropriately describe all the characteristics of tumor even though some of the reported analyses showed achievements [9,10]. Because the prognosis or the treatment response in disease is the result of a complex biological system, it is difficult to identify data types that may significantly contribute.

One solution to the above problem is the use of multi-omics data. Computational power can handle a wide variety of data in parallel, identify truly useful features, combine them and finally create a model that can predict the outcome accurately [8]. Ramazzotti et al. identified a new cancer subtype that was associated with a poor patient outcome, using gene expression, methylation, point mutation and copy number changes [11]. Initially, Chaudhary et al. developed a pipeline to predict a patient’s prognosis using autoencoder for input omics dimension reduction and applied a machine learning model to the test dataset to predict it in liver cancer [12]. They used DNA methylation, miRNA and mRNA as omics datasets with clinical information. Later, we developed a pipeline only using miRNA and mRNA with clinical information to predict lung cancer patient prognosis and identified five genes whose expression levels were associated with patient survival [13]. The effectiveness of autoencoder for dimensionality reduction in multi-omics analysis has recently been reported by many groups [12,13,14]. Supplementary Table S1 summarizes the advantages and disadvantages of single omics and multi-omics analyses.

Although multi-omics data analysis has great potential in biomedical fields, there are some limitations in previous research. For example, integration of many omics datasets into a multimodal analysis is still technically challenging; technical improvements are always required. In addition, with current technology, it is not easy to precisely identify which factors regulate clinical outcomes among multi-omics data. Moreover, even if we successfully build a model that predicts clinical outcomes very accurately using multi-omics data, it is difficult to apply it to clinical practice. This is because it is not feasible to perform all omics analyses routinely in clinical practice in terms of cost performance. Furthermore, there have been few reports on the comparison between single omics analysis and multi-omics analysis, so scientific verification is needed. In particular, it is pretty important to verify the importance of multi-omics analysis based on scientific evidence.

In the present study, we used unsupervised machine learning techniques to build a model for predicting the prognosis of lung cancer patients using six different multi-omics datasets from TCGA. Also, we investigated the possibility of using the model to accurately predict the prognosis of patients with lung cancer using a single omics dataset to aim for clinical applications. As a result, we obtained several important tips for the prognostic prediction of lung cancer patients using multi-omics datasets.

## 2. Materials and Methods

### 2.1. TCGA Dataset

**Figure 1:**
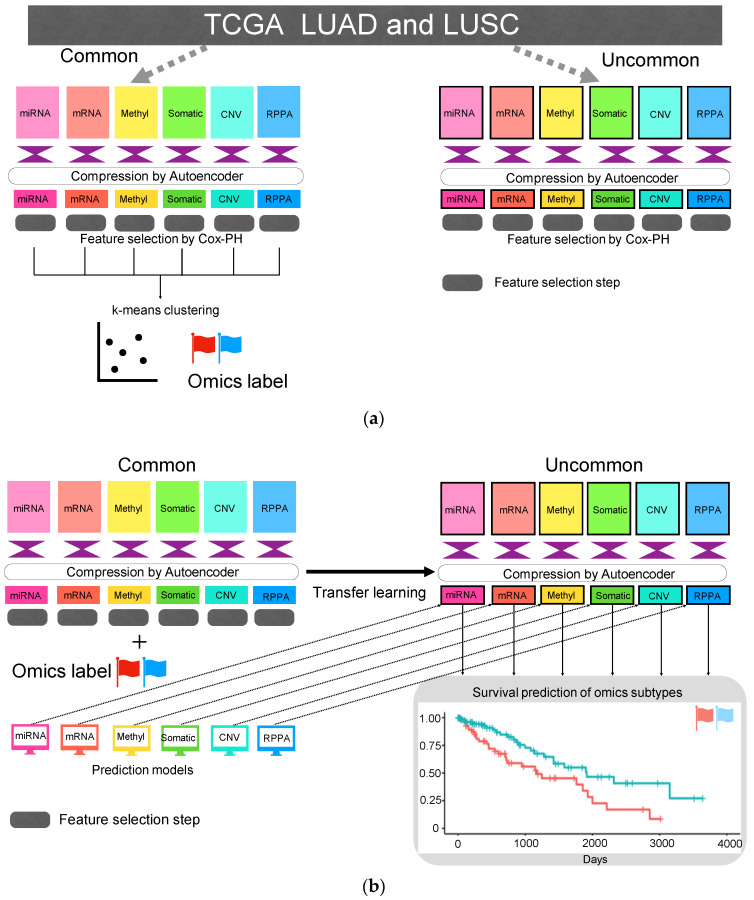
Overall workflow of the study. (**a**) Detecting integration survival subtypes in non-small cell lung cancer (NSCLC) from six categorical multi-omics data in The Cancer Genome Atlas (TCGA). An autoencoder and unsupervised learning technique were used. (**b**) Prediction of integration survival subtypes using only one categorical data and the validation of the model using uncommon data.

The overall data workflow is shown in Figure 1. We obtained multi-omics LUAD and lung LUSC datasets from TCGA portal (https://tcga-data.nci.nih.gov/tcga/) using TCGA-Assembler 2 in March 2020 [15]. Multi-omics data consisted of RNA sequencing data (mRNA; preprocessed using DownloadRNASeqData and ProcessRNASeqData functions), miRNA sequencing data (miRNA; defined using human reference genome 19 and miRBase version 20 [http://www.mirbase.org/]; preprocessed using DownloadmiRNASeqData and ProcessmiRNASeqData functions), DNA methylation data (Methylation; JHU-USC Human Methylation 450, Level 3; preprocessed using DownloadMethylationData and ProcessMethylation450Data functions), copy number variation (CNV; defined using human reference genome 19; preprocessed using DownloadCNAData and ProcessCNAData functions), somatic mutation DNA sequencing data (somatic mutation; preprocessed using DownloadSomaticMutationData and ProcessSomaticMutationData functions) and RPPA data (RPPA; preprocessed using DownloadRPPAData and ProcessRPPADataWithGeneAnnotation functions). For the DNA methylation, we selected CpG islands within 1500 base pairs (bp) ahead of transcriptional start sites (TSS) and used the mean of their methylation values. Clinical data were also downloaded from TCGA portal using TCGA-Assembler 2 and preprocessed using DownloadClinicalData and DownloadBiospecimenClinicalData functions. Patients with a follow-up period longer than 1 day and shorter than 10 years were used in the study. This is intended to use as much patient data as possible. However, if the follow-up period is shorter than 1 day, it means that the patient is not followed up, so those cases were excluded. We constructed a data matrix given a set of sample IDs in rows and gene symbols in columns (Entrez gene id or miRNA name). Data were preprocessed according to the previous publications with following changes [12,13]. First, columns having zero values were removed. We then standardized each data matrix for each row (sample ID) with the exception of the somatic mutation data. This is because the values of somatic mutation data are either one or zero (mutation exists or does not). It is important to note that in previous reports [12,13], the data were merged and then standardized with sample IDs but in this study, the single omics data were standardized with sample IDs. When more than one column has an identical name (for example, gene expression from single gene was observed in two different probes), the columns were merged and the mean value was used. In the case of somatic mutation data, it was set to 1 if mutation exists. The sample IDs common to all data types were defined as common IDs and the other IDs as uncommon IDs. Hence, the data were classified into two datasets as common and uncommon accordingly. The common IDs consisted of 278 cases of LUAD and 205 cases of LUSC (Table 1). To provide an example for better understanding, miRNA_common and methylation_uncommon data sizes are 483 × 217 and 266 × 19,899, respectively.

### 2.2. Autoencoder

Dimensional reductions of the data were implemented by an autoencoder [12,13]. The settings of the autoencoder hyperparameters essentially followed our previous report [13]. The difference of our previous study is that we performed the autoencoder by each omics data (one type of data). The autoencoder codes were written in Python package Keras (https://keras.io) and consisted of three densely connected neural net layers (500, 100 and 500 nodes) with two dropout layers (dropout rate was 0.5) placed between the neural net layers. The parameters of the densely-connected layers were defined as follows: activation function was tanh, L2 regularization function applied to the kernel weights was 0.001 and L1 regularization function applied to the output of the layer was 0.0001. Stochastic gradient descent was selected as the optimizer and the learning rate was set at 0.01 with a decay of 1.0 × 10^−6^. The loss function of the autoencoder was mean square error and the autoencoders were trained for 150 epochs.

Six types of omics data were used in the autoencoder model. For all common data category, the data were split for training and validated in the ratio of 80:20. Uncommon data were not used for training and validation, used only for predicting Class ID (refer to Section 2.5). After training for 150 epochs, all common and uncommon data were dimensionally reduced using the trained autoencoders. In order to equalize the effect after compression, we compressed all data types into a unified feature set of 100.

### 2.3. Feature Selection and k-Means Clustering

Applying the autoencoders to the six types of data matrixes independently, the number of the input features in each matrix was reduced to 100. Of note, the selected features were combined for further studies. This was achieved by following steps. First, the six reduced data matrixes were standardized by scaling. Second, the statistically significant features associated with patient survival were identified by Cox proportional-hazards (Cox-PH) models from the scaled matrixes. Next, the selected features were merged according to the sample ID to get the final data matrix (hereinafter, the matrix is called ‘omics matrix’). To select the clinically meaningful features from the compressed 100 × 6 features, a univariate Cox-PH model was analyzed by using R survival package.

Clusters of common sample IDs were created using k-means clustering method from omics matrix. To obtain the appropriate number of clusters, elbow-method was first used [16]. Then, the Calinski-Harabasz criterion and the Silhouette index were used to obtain the number for best clustering [17,18]. Finally, the value of K, the appropriate number of clusters was clustered by k-means clustering and visualized using the t-distributed stochastic neighbor embedding (t-SNE) [19]. The labels obtained by the above process were referred as Cluster ID. Searching for the appropriate K and t-SNE clustering were performed by using python scikit-learn library.

### 2.4. Machine Learning Models that Predict Cluster ID

Machine learning models that predict the Cluster ID were made from separated omics matrixes, including each of the six data type. The algorithm used was logistic regression with the following parameters; penalty is L2 and C as the inverse regularization strength is 1.0. The performance of the models was evaluated by measuring the area under curve (AUC) using test portion of each omics matrix (the train-test split with an 80:20 ratio). The above procedure was repeated five times and the machine learning models were evaluated by the average AUCs.

### 2.5. Predict Cluster ID Using Compressed Uncommon Data

For further evaluation of the machine learning model described in Section 2.4, uncommon data was used. Six types of uncommon data were dimensionally reduced using the trained autoencoders (refer to Section 2.2). Using selected features (refer to Section 2.3) from the compressed uncommon data as input, the machine learning models predicted the Cluster ID. The inferred Cluster ID was evaluated using Kaplan-Meier analysis (refer to Section 2.7).

### 2.6. Identification of the Proteins Associated with Cluster ID

To find out which proteins were closely related to Cluster ID, we built two machine learning models (XGBoost and LightGBM) using uncompressed common RPPA data. These two models are gradient boosted decision trees frameworks and typically superior to other algorisms in terms of performance, in particular speedup [20,21]. The parameters of XGBoost were as follows: the learning rate was 0.2; the maximum depth of the trees was 3; and the subsample ratio of the training instance was 0.5. The parameters of LightGBM were as follows: the maximum number of bins in which feature values would be bucketed was 256; the learning rate was 0.05; and the number of leaves was 10. The models were evaluated by using AUC (refer to Section 2.7). Finally, the models were explained using SHapley Additive exPlanations (SHAP) [22], which is a game theoretic approach that can interpret the output of machine learning models.

### 2.7. Statistical Analysis

To evaluate the validity of the Cluster ID, Kaplan-Meier analysis was performed. The survival analysis was performed using the R survival package and the survival curves were drawn. With regard to the correlation analysis, we used the corrcoef function NumPy library for Python to compute the Pearson’s correlation coefficient (https://numpy.org/doc/stable/reference/generated/numpy.corrcoef.html) and the pointbiserialr function scipy stats library to compute point biserial correlation coefficient (https://docs.scipy.org/doc/scipy-0.14.0/reference/generated/scipy.stats.pointbiserialr.html). As for evaluation of a statistical relationship between integration survival subtypes and tumor subtypes, we conducted a Chi-square test of independence using the chi2_contingency function from scipy stats library (https://docs.scipy.org/doc/scipy/reference/generated/scipy.stats.chi2_contingency.html). To evaluate the machine learning models that predict the integration survival subtypes made from uncompressed RPPA common dataset, the Sklearn package in Python was applied to calculate the AUC of the receiver operating characteristic (ROC) curve (https://scikit-learn.org/stable/modules/generated/sklearn.metrics.roc_curve.html).

## 3. Results

### 3.1. Unsupervised Approach for Obtaining Clinically Meaningful Subtypes

Clinical data and multi-omics data including six different types of categorical data (miRNA, mRNA, methylation, CNV, somatic mutation and RPPA) were obtained from the TCGA LUAD and LUSC datasets (Figure 1). Data were separated on the basis of the sample IDs as follows: common and uncommon IDs. Common IDs included all six types of multi-omics data and additional IDs were categorized as uncommon IDs. A total of 483 common IDs were recovered and the number of uncommon IDs varied depending on the data type (Table 1).

Autoencoders were applied to the six types of categorical omics data and their dimensions were reduced to 100 each. To select features significantly associated with patient survival, univariate Cox-PH regression was carried out with the reduced dataset. The feature selection criteria were set as follows: (1) log-rank test *p* < 0.01 or (2) 0.01 < log-rank test *p* < 0.05 and top three *p*-values within each category. Consequently, a total of 29 features were selected, which consisted of 12 mRNAs, 3 miRNAs, 3 methylations, 5 CNVs, 3 somatic mutations and 3 RPPA features (Table 2). The data were merged into a single matrix, referred as the omics matrix.

**Figure 2:**
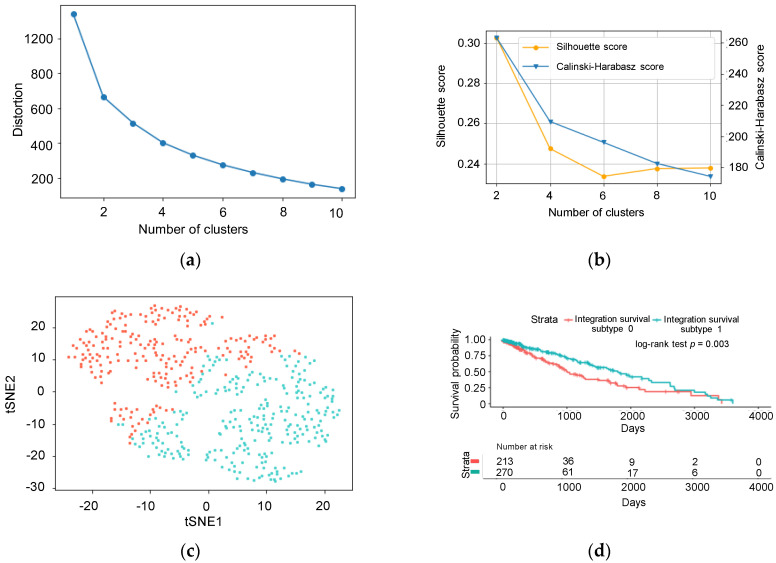
Prediction of the cluster number and k-means clustering. (**a**) Result of the elbow method. The *x*-axis shows the number of clusters; the *y*-axis shows the distortion score. (**b**) Result of the Calinski-Harabasz index and Silhouette Coefficient. The *x*-axis shows the number of clusters; the *y*-axis shows the Silhouette score or Calinski-Harabasz score. (**c**) Visualization of the k-means clustering by t-SNE. (**d**) Kaplan-Meier survival curves of integration survival subtypes.

Next, we aimed to determine the appropriate number of clusters using the omics matrix. The optimal clustering number was determined using the Calinski-Harabasz criterion and Silhouette index (Figure 2a,b after a rough estimation using the elbow method. All previously mentioned methods indicated that the optimal number of clustering was two. Figure 2c shows the result of the k-means clustering visualized by t-SNE. The inferred labels from the k-means clustering were referred to as Cluster ID. The survival analysis showed a significant difference in survival rates using the Cluster IDs (log-rank test: *p* = 0.003, Figure 2d) and the subtypes clustered were referred to as integration survival subtypes. The number of patients belonging to integration survival subtype 1 (longer survival) and integration survival subtype 0 (shorter survival) was 270 and 213, respectively. There was no significant relationship between the tumor histopathological subtype (LUAD or LUSC) and the integration survival subtypes (Chi-square test of independence: *p* = 0.94), indicating that our model could predict patient survival, independent of tumor subtypes, including both LUAD and LUSC.

### 3.2. Predicting Integration Survival Subtypes Using Compressed Categorical Datasets

**Figure 3:**
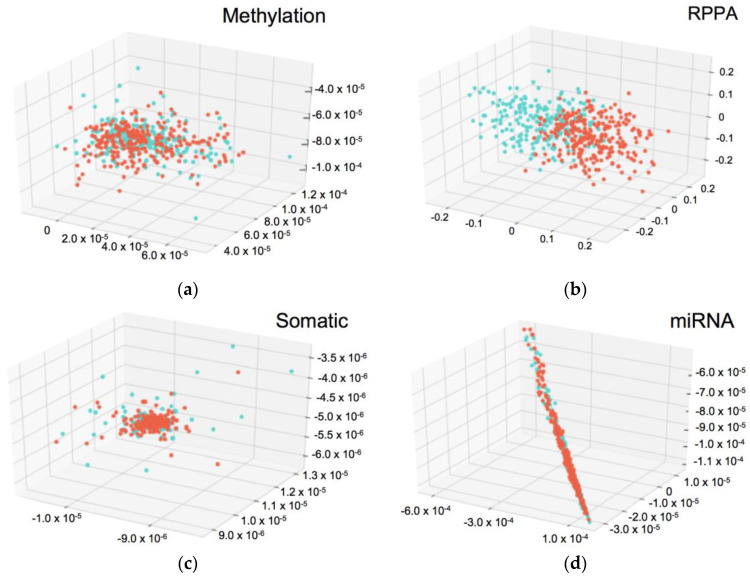
3D-scatter plots of compressed common ID data belonging to one category. Each axis represents the data values and the color shows Cluster ID. (**a**) Methylation common data. (**b**) reverse phase protein array (RPPA) common data. (**c**) Somatic mutation common data. (**d**) miRNA common data. The Cluster ID are not separated in (**a**,**c**,**d**). In (**b**), the Cluster ID were separated clearly.

We developed machine learning models that would predict integration survival subtypes using compressed common ID data belonging to one category (e.g., miRNA common, Figure 3). First, the omics matrix was divided by each category. For example, sizes of mRNA and miRNA of the omics matrix were 483 × 12 and 483 × 3, respectively. Figure 3 shows the distributions of divided omics matrices that have three features. On the basis of the distribution, the RPPA portion of the omics matrix appears to predict integration survival subtypes. CNV and mRNA could not be visualized in the 3D plot as more than three features were available after Cox-PH selection (Table 2). Next, six logistic regression models were developed from each omics matrix. Finally, the model performance was evaluated using AUC (Table 3). Only one machine learning model that was trained on the RPPA omics matrix could accurately predict the integration survival subtypes (AUC = 0.99).

### 3.3. Validation Using Uncommon RPPA Datasets

**Figure 4:**
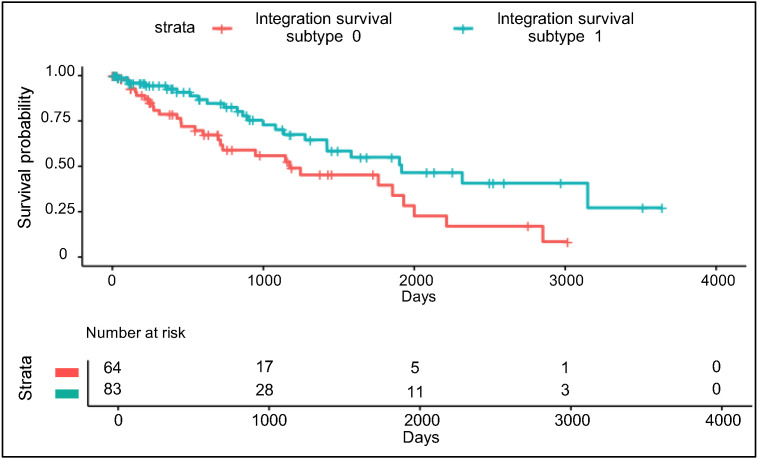
Kaplan-Meier survival curve of the RPPA uncommon dataset using the integration survival subtypes.

The validity and the predictability of the integration survival subtypes were assessed using RPPA_uncommon data. RPPA_uncommon data were compressed using the trained autoencoder (refer to Section 3.1). The same features identified in Section 3.1 were selected from the compressed RPPA_uncommon features. Next, using these features as input, the subtypes were predicted by the logistic regression model trained on the RPPA part of the omics matrix (refer to Section 3.2). Patients were labelled as either integration survival subtype 1 (*n* = 83) or integration survival subtype 0 (*n* = 64) and there were significant differences in survival between the two subtypes (log-rank test: *p* = 0.012, Figure 4). There was no relationship between the inferred integration survival subtypes and the histological subtypes (Chi-square test of independence *p* = 0.61), suggesting that our model predicted patient survival independent of histological subtypes.

### 3.4. Comparison of Integration Survival Subtypes and RPPA Survival Subtypes

We then built another machine learning model to predict the RPPA survival subtype by creating a directory from the RPPA common data. The aim was to validate the usefulness of the integration survival subtype by comparing the integration survival subtypes with the RPPA survival subtypes; we followed the same procedure as in Section 3.1, Section 3.2 and Section 3.3. First, we performed univariate Cox-PH regression using RPPA common data to select proteins significantly associated with patient survival. The top selected proteins are shown in Supplementary Table S2. ERRFI1, CCND1 and BCL2 were selected using the same criteria as the multi-omics features ((1) log-rank test *p* < 0.01 and (2) 0.01 < log-rank test *p* < 0.05, the top three *p* values within each category). Subsequently, we then performed unsupervised clustering and found survival-related subtypes. The optimal clustering number was two, determined using the Calinski-Harabasz criterion and the Silhouette index (Supplementary Figure S1A,B). The result of k-means clustering visualized by t-SNE is presented in Supplemental Figure S1C. Also we referred to the labels inferred from k-means clustering as RPPA survival subtypes. Survival analysis showed a significant difference in survival rates using the RPPA survival subtypes (log-rank test: *p* = 0.003, Supplementary Figure S2A). A logistic regression model that was trained on selected three protein values from RPPA common data, could predict RPPA survival subtype accurately (AUC = 0.99). Although the logistic regression model predicted inferred RPPA survival subtypes using RPPA uncommon data, there were no significant differences in survival between the two inferred RPPA survival subtypes (log-rank test: *p* = 0.9, Supplementary Figure S2B)

### 3.5. Insight into the Proteins Associated with Integration Survival Subtypes

**Figure 5:**
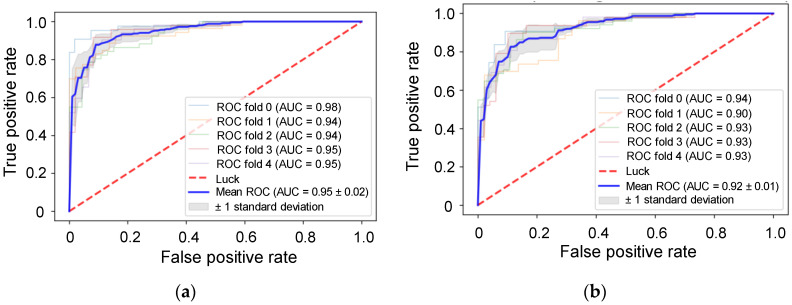
Receiver operating characteristic (ROC) analysis for evaluation of the machine learning models that predict the integration survival subtypes using uncompressed RPPA common datasets. ROC curves of XGBoost (**a**) and LightGBM (**b**).

**Figure 6:**
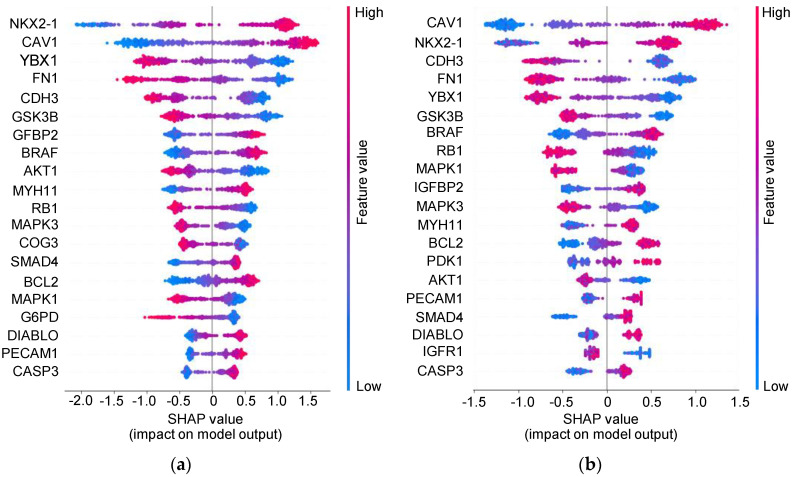
SHapley Additive exPlanations (SHAP) summary plot. (**a**) The plot shows the SHAP value of XGBoost magnitudes across all samples. The color represents the feature values (red represents high and blue represents low). (**b**) The plot shows the sum of SHAP value of LightGBM.

We suspected that RPPA was a good indicator of the integration survival subtype. Therefore, we decided to use common_RPPA data and implemented XGBoost and LightGBM to predict integration survival subtypes. Machine learning models that use gradient boosting, including XGBoost and LightGBM, have high success in clinical settings. Here, the AUC of XGBoost and LightGBM was 0.95 ± 0.02 and 0.92 ± 0.01, respectively (Figure 5). We attempted to interpret the developed models using SHAP. A summary is available in Figure 6. The important proteins identified by each model were nearly identical. The top five proteins predicted by two models, NKX2-1, CAV1, CDH3, FN1 and YBX1, were exactly the same. The relationship between the expression of these five proteins and the prognosis of the cancer patients was almost consistent with that described in previous reports [23,24,25,26,27,28]. CAV-1 and YBX-1 are generally negative predictors of NSCLC outcomes [24,25]. FN1 seems to be associated with cisplatin resistance and over-expression of CDH3 correlates to a poor prognosis in carcinomas of the breast, prostate, ovary, colon and stomach [29,30].

NKX2-1 has been shown to play a critical role in lung development, lung cancer differentiation and morphogenesis, particularly in LUAD [23]. Meanwhile, our analysis showed no statistically significant differences in expression between LUAD and LUSC in both RPPA and mRNA levels (Supplementary Figure S3, Welch’s *t*-test: *p* = 0.143 [RPPA] and *p* = 0.073 [mRNA], respectively). Several studies focusing on the role of NKX2-1 in lung cancer prognosis have already been examined. Most of the studies have shown a positive correlation between NKX2-1 over-expression and survival, which is consistent with our findings (Figure 6).

**Figure 7:**
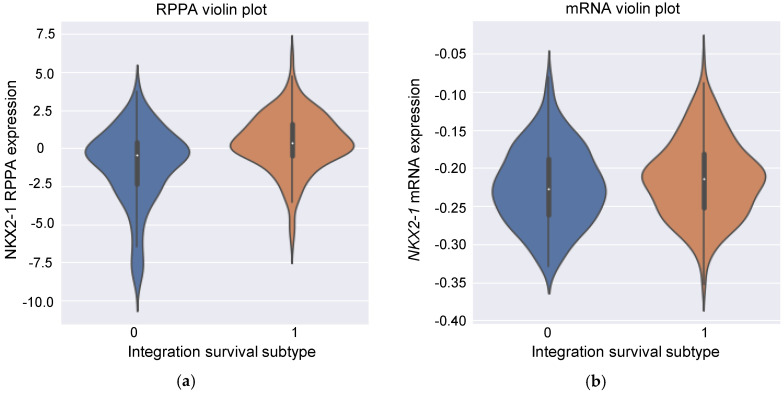
Relationship between Cluster ID and NKX2-1 expression levels. (**a**) Relationship between NKX2-1 RPPA expression levels and integration survival subtypes. x-Axis shows the integration survival subtype and Y-axis shows the value of NKX2-1 RRPA expression levels that are standardized against row (sample ID). (**b**) Relationship between *NKX2-1* mRNA expression levels and integration survival subtypes. x-Axis shows integration survival subtype and *y*-axis shows the value of *NKX2-1* mRNA expression levels that are standardized against row (sample ID).

Our analysis could explain conflicting results not yet fully addressed. NKX2-1 expression is generally thought to be associated with a good prognosis, although some studies have reported the opposite [23,31,32,33,34,35,36,37,38,39,40,41,42,43,44]. Yoon et al. showed that NKX2-1-positive circulating tumor cells (CTCs) were specific to NSCLC patients and the CTCs negatively correlated with the survival [44]. There were two major differences between their study and other studies. First, Yoon et al. focused on CTCs, whereas other studies, including ours, used tumor resection specimens. Second, they used nested real-time RT (reverse transcription)-PCR assay detecting mRNA. Other studies used immunostaining, which detects protein expression. Then we investigated the relationship between RNA expression and protein expression levels. As shown in Figure 7a, there was a weak positive correlation between NKX2-1 RPPA expression levels and the integration survival subtypes because high levels of NKX2-1 RPPA expression are more likely to be classified as integration survival subtype 1 (point biserial correlation coefficient: *r_pb_* = 0.323). However, there was no correlation with *NKX2-1* mRNA expression levels: high levels of *NKX2-1* mRNA expression tended to be labelled as integration survival subtype 1, whereas low levels of *NKX2-1* mRNA expression tended to be labelled as integration survival subtype 0 (Figure 7b, point biserial correlation coefficient: *r_pb_* = 0.064). This tendency is concordant with that seen in Figure 6. Meanwhile, there was a statistically significant difference in the expression of both NKX2-1 RPPA and mRNA between integration survival subtype 0 and 1 (Welch’s *t*-test: *p* < 0.001 [RPPA], *p* = 0.025 [mRNA], respectively). Importantly, there was no correlation between NKX2-1 RPPA expression levels and *NKX2-1* mRNA expression levels (Supplementary Figure S4, Pearson’s correlation coefficient: *r* = 0.102). Recently, it has become evident that mRNA levels are not sufficient to predict protein levels and our results are consistent with the previous report [45]. Hence, it is possible to explain the different results obtained by other groups and those observed in the present study in the context of NKX2-1 [44].

## 4. Discussion

Omics data analysis has potential for predicting patient outcomes such as prognosis and drug resistance. Multiple analyses using various categorical data have been conducted to understand the nature of cancer. The analysis of each layer is valuable on its own but there are limitations to using only one categorical dataset. A single omics data-derived model has a relatively small power of correcting the results, noises and/or missing values within the omics data. No matter how accurately developed the model, we would not be able to overcome the disadvantage of using one categorical data. Therefore, the use of multi-omics data is a possible and reasonable approach that could minimize the individual bias of each data type through integration of the various types of data; our results in the present study support this concept. RPPA data were useful for predicting integration survival subtypes and integrated survival subtypes could predict survival of patients with lung cancer robustly. But RPPA survival subtypes, made from RPPA data directly, could not predict it. These two subtypes were also different in the proteins that were critical for each subtype. This difference could be caused by the noise and bias with in the RPPA data. In other words, our multi-omics data integration procedure may relive the noise and bias.

Another drawback to using only one platform is a limitation of the analysis itself. For example, it would be difficult to predict the size of a tumor using CNV. Instead, a medical imaging method such as computed tomography (CT) would be more accurate for this purpose. However, in reality it is more complex. In fact, we do not know what the best omics data would be to perfectly predict patient outcomes, since the physiological responses of human bodies are so diverse. To illustrate, one analysis may show a positive response and another may report a negative response. This makes it quite challenging to predict the exact outcomes of interest. Therefore, in this study, we used as many categories of multi-omics data as possible to predict patient survival and to identify genes related to the subtype. Since the appropriate data type required for our aim is unknown, the use of multiple categorical data should be advantageous. Although we discussed the benefit of using as many categories of data as possible, finding meaningful features from tens of thousands of inputs from multi-omics data is a technical barrier. For this reason, we implemented the autoencoder model to predict patient survival from only one categorical RPPA dataset and the RPPA was further used to examine the molecular insight by using gradient boosting machine learning models followed by SHAP analysis. With our previous and current study, we demonstrated that the autoencoder combined with machine learning models for deeper insight for molecular biology is one of the most powerful pipelines available to investigate cancer biology from a different perspective [13]. We also showed the possibility of predicting patient survival with one categorical dataset and potentially related genes in integration survival subtypes. However, it should be noted that there is still heavy risk involved when relying on the results obtained from a single omics dataset.

There are several problems to overcome in multi-omics analysis. One such issue is generally called as the “Large *p* Small *n* Problems”; there are far more parameters or features in input dimension compared to the number of sample [46]. This would result in overfitting, leading to a poor performance. To avoid this, unsupervised algorithms could be applied; however, this would ignore known factors such as tumor type. Hence, effectively selecting features or reducing data dimensions is essential for using big multi-omics data and thus, the autoencoder is one of the key techniques to reduce the dimension. Autoencoders aim to reconstruct the original-input using a combination of nonlinear functions and after training, the bottleneck features can be used as the latent expression of the original input [12,13]. However, further study is needed to determine the appropriate setting of autoencoder. For example, we uniformly set the number of features after autoencoder compression to 100. This operation will have an equal impact after compression but data with more features before compression must lose more information than data with fewer features. In addition, the 3D scatter plot of miRNAs showed a straight line, implying that the autoencoder setting is not appropriate for miRNA data (Figure 3c). In any case, a more detailed study is needed to determine whether the settings of the autoencoder should be changed depending on the type of data and if so, what settings should be used.

Another problem encountered while incorporating machine learning models trained by multi-omics data is the expense. Costs of examinations using high-throughput technology in hospitals or laboratories have been decreasing annually; however, it might not be practical to perform multiple categories of sequencing or microarray analyses routinely for all new patients. Here, we predicted the clinical subtypes from omics analysis using only one categorical RRPA dataset despite drawbacks of using only one categorical data. Our developed model could successfully predict the patient prognosis using the RPPA data on new lung cancer patients. This has implications for decision-making tools and our pipeline may reduce the cost of examination through elimination of unnecessary surveys and acceleration of routine omics analysis benefitting precision medicine [47].

The fundamental problem encountered is the difficulty in resolving the relationship between compressed features and biological meanings. There have been reports of biologically interpretable deep learning frameworks but they achieved limited success [48]. In this study, our platforms aimed to interpret the features though autoencoders and partially with machine learning models using SHAP (Figure 6).

There are some limitations in our study. First, we used unsupervised machine learning algorithms to create Cluster IDs but unsupervised learning techniques tend to identify only those with the strongest signals. Therefore, our integration survival subtypes may not be truly multi-omics-derived. Second, we set the number of features to 100 after uniformly compressing them with the autoencoder. This operation has the same effect after compression but data with more features before compression must lose more information than data with fewer features. Further studies are needed to determine the appropriate number of features after compression. Third, we used uncommon datasets as testing dataset. While the procedure for splitting common and non-common data is not arbitrarily, we think that ideally a complete set of all types of data should be prepared and tested.

Nonetheless, we think our pipeline is effective as we have demonstrated its success through identification of genes that are associated with subtypes. Improved methods are ideal moving forward.

## 5. Conclusions

Our study predicted the integration survival subtypes in NSCLC that were independent of the tumors histological types by using six types of multi omics data. The integration survival subtypes were also predicted by using only RPPA data. We validated the models using uncommon data as an independent test dataset. Omics analysis has a huge potential as we shown previously and in this study. We identified at least five proteins of interest (NKX2-1, CAV1, YBX1, FN1 and CDH3) with two different machine learning models that may be associated with lung cancer patient survival. This study offers the benefits of analyzing multi-omics data using the combined approach of deep learning and machine learning methods for predicting prognosis. Our method may be more robust than traditional single omics methods and can predict integration survival subtypes using gradient booster models with genes potentially associated with the subtype, especially in NSCLC patients.

## Figures and Tables

**Table 1:** The summary of common and uncommon data set.

The Number of Samples of Each Data Type			
Data Name	LUAD	LUSC	Total
Common	278	205	483
Clinical_uncommon	197	262	459
mRNA_uncommon	190	262	452
miRNA_uncommon	125	103	228
RPPA_uncommon	54	93	147
CNV_uncommon	190	259	449
Somatic mutation_uncommon	193	249	442
Methylation_uncommon	135	131	266

**Table 2:** The summary of data used.

The Number of Features in Each Step			
Data Type	Before Compression	After Compression by Autoencoder	After Feature Selection by Cox-PH
mRNA	13,049	100	12
miRNA	217	100	3
RPPA	150	100	3
CNV	14,786	100	5
Somatic mutation	18,977	100	3
Methylation	19,899	100	3

**Table 3:** Area under curve (AUC) of logistic regression models for predicting the survival subtypes using compressed data.

Data Type	AUC
mRNA	0.57 ± 0.05
miRNA	0.61 ± 0.07
RPPA	0.99 ± 0.00
CNV	0.43 ± 0.04
Somatic mutation	0.50 ± 0.07
Methylation	0.55 ± 0.05

## Supplementary Materials

The following are available online at https://www.mdpi.com/2218-273X/10/10/1460/s1, Figure S1: Prediction of the cluster number and k-means clustering using RPPA common data. Figure S2: Kaplan-Meier survival curve of the RPPA common and uncommon dataset. Figure S3: Relationship between histological subtypes of lung cancer and NKX2-1 expression levels. Figure S4: Scatter plot analysis of NKX2-1 mRNA and RPPA expression levels. Table S1: The summary of single and multi-omics analysis. Table S2: The summary of selected protein strongly related with survival using Cox-PH regression model in common RPPA data.
