# Supplementary material for: Predicting Deep Learning Based Multi-Omics Parallel Integration Survival Subtypes in Lung Cancer Using Reverse Phase Protein Array Data
Source: Biomolecules. 2020 Oct 19;10(10):1460. doi: 10.3390/biom10101460 (PMC7603376; doi:10.3390/biom10101460)
Supplement: Supplementary file 1 [file biomolecules-10-01460-s001.pdf]

Supplementary information

Predicting Deep Learning Based Multi-Omics Parallel Integration Survival Subtypes in Lung Cancer Using Reverse Phase Protein Array Data

The file contains

Supplementary Figures 1 – 4

Supplementary Tables 1 – 2

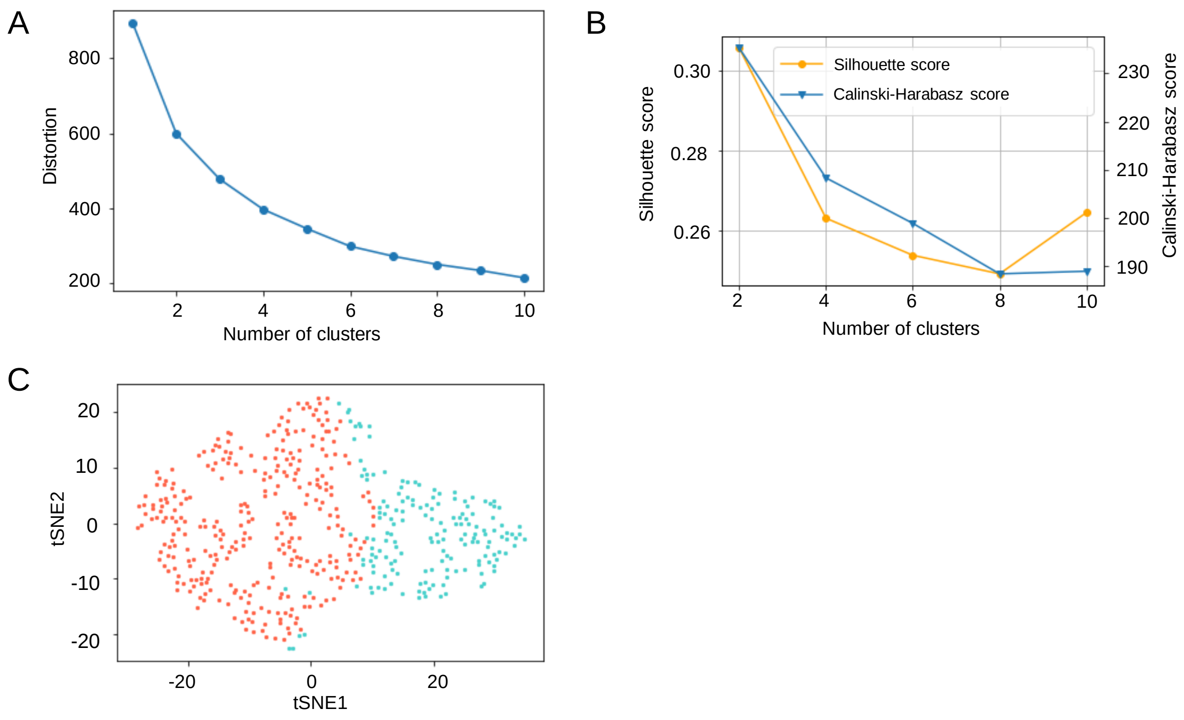

Supplementary Figure S1. Prediction of the cluster number and k-means clustering using RPPA common data. (A) Result of the elbow method. The *x*-axis shows the number of clusters; the *y*-axis shows the distortion score. (B) Result of the Calinski-Harabasz index and Silhouette Coefficient. The *x*-axis shows the number of clusters; the *y*-axis shows the Silhouette score or Calinski-Harabasz score. (C) Visualization of the k-means clustering by t-SNE.

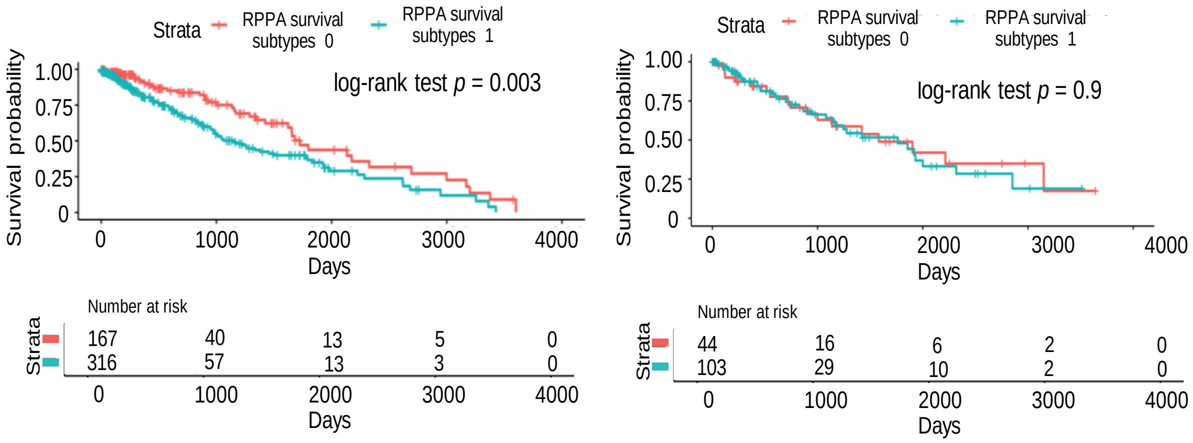

Supplementary Figure S2. Kaplan-Meier survival curve of the RPPA common and uncommon dataset. (A) RPPA common dataset using the RPPA survival subtypes. (B) RPPA uncommon dataset using the inferred RPPA survival subtypes.

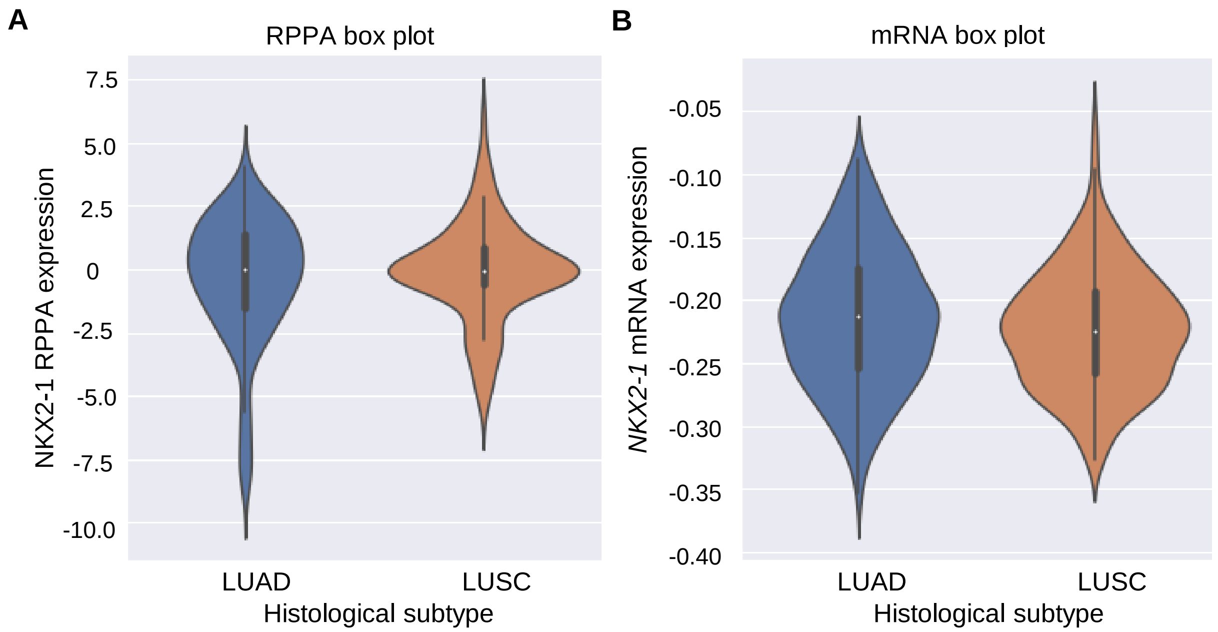

Supplementary Figure S3. Relationship between histological subtypes of lung cancer and NKX2-1 expression levels. (A) Relationship between NKX2-1 RPPA expression levels and histological subtypes of lung cancer (LUAD or LUSC). *x*-Axis shows the histological subtypes of lung cancer, and *y*-axis shows the value of NKX2-1 RRPA expression levels that are standardized against row (sample ID). (B) Relationship between *NKX2-1* mRNA expression levels and histological subtypes. *x*-Axis shows histological types of lung cancer, and *y*-axis shows the value of *NKX2-1* mRNA expression levels that are standardized against row (sample ID).

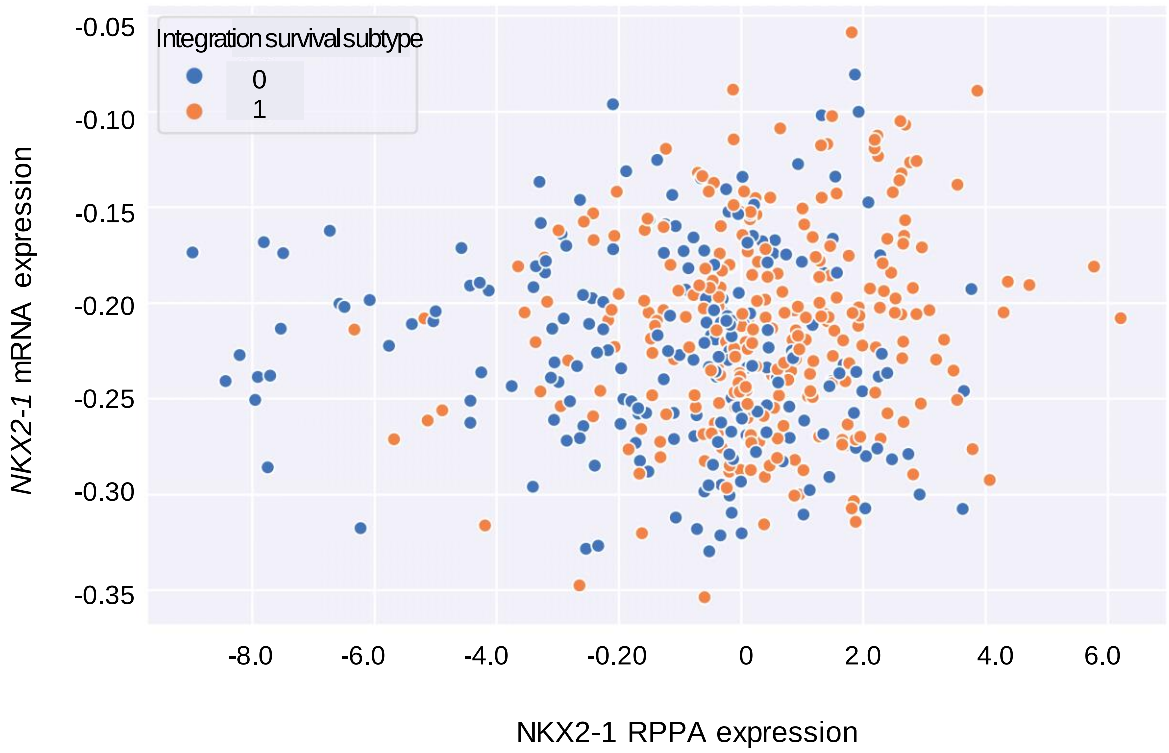

Supplementary Figure S4. Scatter plot analysis of NKX2-1 mRNA and RPPA expression levels.

Supplementary Table S1. The summary of advantages and disadvantages regarding single omics and multi-omics analyses.

Items	Single omics analysis	Multi omics analysis
Robustness for noise	Weak	Strong
Amount of information	Small	Large
Cost	Low	High
Analysis method	Well developed	Developing now

Supplementary Table S2. The summary of selected protein strongly related with survival using Cox-PH regression model in common RPPA data.

**Protein**	**Log-rank *p* value**
ERRFI1	0.017
CCND1	0.018
BCL2	0.024
KDR	0.031
PRKCA	0.035
RAB25	0.042
FN1	0.046
CLDN7	0.047
G6PD	0.047
PECAM1	0.048
